# Perception of Good Death and Death Preparation Among Community-Dwelling Older Adults in South Korea

**DOI:** 10.1093/geroni/igaf122.2317

**Published:** 2025-12-31

**Authors:** Bomgyeol Kim, Hun Kang, Seongmi Choi, JaeWon Hyun, JiYeon Choi

**Affiliations:** Yonsei University College of Nursing, Seoul, Republic of Korea; Yonsei University, Seoul, Republic of Korea; Daegu Catholic University, Daegu, Republic of Korea; Yonsei University, Seoul, Republic of Korea; Yonsei University, Seoul, Republic of Korea

## Abstract

As societies age, preparing for a good death is becoming a critical public health concern. This study investigated how perceptions of a good death shape engagement in death preparation among community-dwelling older adults in South Korea. We conducted a secondary analysis of the 2023 National Survey of Older Koreans. The final sample included 9,140 older adults (≥65 years), excluding proxy responses and severe cognitive impairment (K-MMSE∼2 ≤17). Death preparation was assessed based on engagement in eight specific activities, categorized into practical and conceptual death preparation. Perceptions of a good death were measured using a five-item scale evaluating key end-of-life aspects. Participants were predominantly female (60.0%) and in the 65-74 -year-old group (60.7%). Among participants, 51.3% engaged in practical death preparation, while 5.5% in conceptual death preparation. Results of multiple logistic regression revealed that those who valued organizing personal affairs (aOR = 1.12, 95% CI = 1.05–1.21), minimizing the burden on family (aOR = 1.18, 95% CI = 1.11–1.26), and preferring to die at home (aOR = 1.06, 95% CI = 1.01–1.12) were more likely to engage in practical death preparation. Conceptual death preparation was most strongly associated with preferring to die at home (aOR = 1.52, 95% CI = 1.35–1.70), whereas being surrounded by loved ones was negatively associated (aOR = 0.77, 95% CI = 0.67–0.88). These findings highlight the need for tailored policies and community-based interventions to help older adults align end-of-life preferences with preparation, promoting meaningful aging and a dignified death.

